# Rapid Fabrication of Capillary-Sized Microchannels in Collagen Hydrogel via Thermally Responsive Gelatin Microfiber Templates

**DOI:** 10.3390/ma19173630

**Published:** 2026-08-26

**Authors:** Takayuki Takei, Momoka Nakamura, Ko Nishimura, Saki Kobaru, Yoshihiro Ohzuno, Masahiro Yoshida

**Affiliations:** Department of Chemical Engineering, Graduate School of Science and Engineering, Kagoshima University, 1-21-40 Korimoto, Kagoshima 890-0065, Japan; k2640129@kadai.jp (M.N.); k7787358@kadai.jp (K.N.); inamine@eng.kagoshima-u.ac.jp (S.K.); ohzuno@cen.kagoshima-u.ac.jp (Y.O.); myoshida@cen.kagoshima-u.ac.jp (M.Y.)

**Keywords:** capillary, wet spinning, microfiber, sacrificial template, collagen hydrogel, tissue engineering

## Abstract

**Highlights:**

- Physically crosslinked gelatin microfibers (approximately 10 μm in diameter) were fabricated via a unique wet-spinning technique.
- Gelatin microfibers served as a thermal sacrificial template.
- Capillary-sized microchannels were rapidly established within 1 h.

**Abstract:**

Engineering volumetric three-dimensional tissues requires the rapid establishment of dense, capillary-like microchannels to ensure adequate oxygen and nutrient supply while preventing hypoxic cell necrosis. Sacrificial microfiber templating approaches using lower critical solution temperature (LCST) polymers cannot employ type I collagen hydrogels—a biologically ideal extracellular matrix—because LCST fiber dissolution in cold collagen solutions precedes matrix gelation owing to thermodynamic mismatch. Here, we present a proof-of-concept strategy to rapidly fabricate capillary-sized microchannels within collagen hydrogels using thermoresponsive, physically crosslinked gelatin microfibers as sacrificial templates. Three-dimensional gelatin microfibers with capillary-sized diameters (approximately 10 μm) were fabricated via wet spinning and embedded in a type I collagen aqueous solution (4 °C). Open microchannels throughout the collagen matrix were successfully generated within 1 h of sequential thermal incubation (20 °C for collagen gelation and subsequently 37 °C for gelatin thermal dissolution), without cytotoxic chemicals. Active particle flow confirmed channel patency and fluidic continuity. Additionally, heparinized rat blood readily perfused through the channels, and scanning electron microscopy revealed open microchannel cross-sections (diameter: approximately 10 μm). This simple, thermally controlled approach resolves the limited temperature compatibility between collagen matrices and sacrificial microfibers, serving as a promising biofabrication foundation for engineering tissue constructs.

## 1. Introduction

Recent advances in tissue engineering have led to the creation of transplantable artificial tissues for humans, such as cartilage and corneas [1]. Currently, establishing fabrication technologies for thick, three-dimensional (3D) organs with complex functions and high oxygen demands, such as the liver, remains a major challenge [2]. One of the most critical requirements for creating volumetric 3D organs with high oxygen demands is determining how to supply sufficient oxygen and nutrients to all the cells constituting the organ. Individual cells of native organs receive sufficient oxygen and nutrients owing to the dense capillary network of the organ. Similarly, to create transplantable tissue-engineered organs for human use, it is essential to construct a dense capillary-like network within the constructs [3].

To date, various top-down engineering strategies, including sacrificial material templating and 3D bioprinting [4,5,6,7,8,9,10,11,12,13,14,15,16,17,18,19,20,21,22,23], have been proposed alongside traditional bottom-up approaches relying on the spontaneous tube-forming activity of vascular endothelial cells [24,25,26,27,28]. Endothelial self-assembly typically requires several days, during which encapsulated parenchymal cells risk necrotic death owing to prolonged hypoxia. In contrast, sacrificial templating offers a significantly faster route to establish perfusable channels. Among top-down sacrificial strategies, various classes of sacrificial materials have been investigated, including carbohydrate glass, alginate, hydrophobic polymers, and thermoresponsive polymers [6,14,15,16,17,19]. Carbohydrate glass and alginate templates allow the generation of 3D networks, but typically yield channel diameters larger than native capillaries (>100 µm) or require specific chemical leaching conditions [6,17,19]. Hydrophobic polymer microfibers can achieve capillary-scale dimensions (approximately 10 µm) [6,16]; however, their removal requires immersing the entire construct in cytotoxic organic solvents, which precludes pre-encapsulation of viable organ cells. On the other hand, sacrificial microfibers fabricated from lower critical solution temperature (LCST) polymers (e.g., poly(N-isopropylacrylamide) (PNIPAM) and Soluplus^®^) dissolve in aqueous media at lower temperatures without organic solvents [14,15]. Despite these advances, LCST-based strategies cannot employ type I collagen hydrogels—a biologically ideal matrix for cell culture and remodeling. Collagen hydrogels undergo temperature-induced self-assembly upon warming a cold solution (4 °C) to 37 °C. Consequently, when LCST microfibers are immersed in cold collagen solutions, they dissolve prematurely before collagen gelation occurs owing to a fundamental thermodynamic mismatch. To overcome these limitations, we focused on physically crosslinked gelatin as an upper critical solution temperature (UCST) sacrificial template. Gelatin remains in a solid/gelled state at lower temperatures (4 °C) and undergoes a gel-to-sol transition at physiological temperatures (37 °C), presenting a complementary thermal response that matches cold collagen gelation under entirely aqueous, cell-friendly conditions.

**Figure 1:**
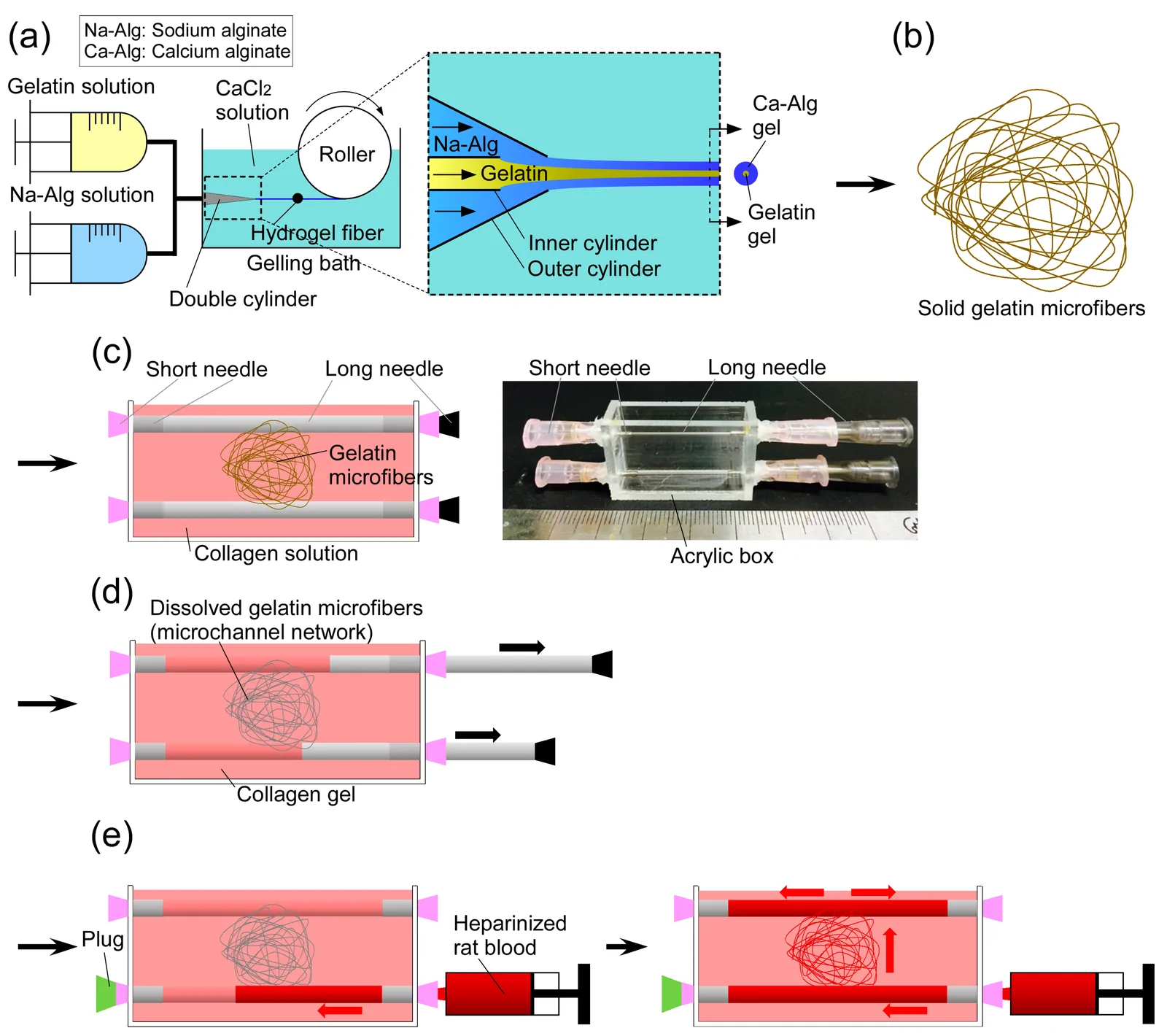
(**a**,**b**) A wet-spinning process to prepare gelatin/calcium alginate (Ca-Alg) hydrogel fibers (**a**) and gelatin microfibers prepared by removal of Ca-Alg hydrogel from the gelatin/Ca-Alg hydrogel fibers and subsequent freeze-drying (**b**). (**c**–**e**) A procedure to fabricate capillary-sized microchannels in a collagen gel. (**c**) Gelatin microfibers are immersed in a cold aqueous collagen solution in the acrylic box shown in the photo on the left. (**d**) The collagen solution was incubated at 20 and 37 °C to achieve gelation of the solution (20 °C) and subsequent dissolution of the microfibers (37 °C, for the formation of microchannels); then, long needles were withdrawn from the box to form straight millichannels connected to the microchannels. (**e**) Heparinized rat blood was infused into the channels to visualize the microchannels.

In this study, we aimed to establish a preliminary proof-of-concept strategy for rapidly fabricating microchannels within collagen hydrogels using thermoresponsive gelatin microfibers. The overall fabrication procedure is outlined in Figure 1. First, physically crosslinked gelatin microfibers similar to native capillaries in diameter were fabricated using our unique wet-spinning technique (Figure 1a,b) [29]. The gelatin microfibers were subsequently immersed in a cold aqueous collagen solution (Figure 1c), followed by incubation at 20 °C and then 37 °C (Figure 1d). This simple temperature elevation triggered thermal gelation of the collagen matrix (20 °C) and thermal dissolution of the gelatin microfibers (37 °C) as a sacrificial template, successfully establishing open microchannels within 1 h. This study focused on validating the fundamental feasibility of this rapid, single-step fabrication process.

## 2. Materials and Methods

### 2.1. Preparation of Gelatin/Ca-Alginate (Ca-Alg) Hydrogel Fibers

Figure 1a shows a schematic of the wet-spinning process used to prepare gelatin/Ca-Alg hydrogel fibers. A coaxial double cylinder, consisting of two stainless-steel needles, was cut at 90° relative to the axial direction. The inner diameters of the tips of both needles were 480 μm. Aqueous solutions of type A gelatin (10% (*w*/*v*)) (Sigma–Aldrich, St. Louis, MO, USA) and sodium alginate (Na-Alg) (1% (*w*/*v*)) (Kimica Co., Tokyo, Japan) were simultaneously extruded from the inner and outer cylinders using syringe pumps, respectively, into 100 mM aqueous calcium chloride (4 °C) in a gelling bath. Both aqueous solutions were maintained at 80 °C for approximately 2.5 h prior to and during extrusion. The Na-Alg solution underwent gelation upon taking up calcium ions from the gelling bath. The gelatin solution gelled as it cooled in the gelling bath via physical crosslinking. The resulting gelatin/Ca-Alg hydrogel fibers were taken up by a roller. The winding speed of the roller was 900 cm/min, and the total flow volume of the gelatin and Na-Alg solutions was fixed at 1.1 mL/min. The ratio of the flow volume of a gelatin solution to the total flow volume of the gelatin and Na-Alg solutions (volume ratio of gelatin solution) was adjusted (0.00225–0.16700) to prepare gelatin hydrogel microfibers of approximately 10 μm in diameter. The mean cross-sectional diameter of the fibers was determined by measuring more than 50 randomly selected sample areas using an optical microscope. Microfiber fabrication was conducted in more than ten independent experimental batches.

### 2.2. Fabrication of Gelatin Microfibers

Residual calcium ions were removed by thoroughly washing the gelatin/Ca-Alg hydrogel fibers with distilled water. The fibers were immersed in a large excess of 100 mM aqueous trisodium citrate (pH 7.4, 4 °C) for 5 days. The trisodium citrate solution was replaced with a fresh solution every day. During this process, the Ca-Alg hydrogel dissolved as calcium ions were chelated with citrate ions and removed from the gel matrix [30]. Removal of the Ca-Alg hydrogel was confirmed using Fourier transform infrared spectroscopy (FT-IR) (in ATR mode, IRT-3000, JASCO Co., Ltd., Tokyo, Japan). The obtained gelatin hydrogel microfibers were immersed in 99% (*v*/*v*) ethanol (4 °C) for 2 h to induce dehydration. Ethanol was replaced with fresh ethanol every 30 min. The gelatin microfibers were then immersed in tert-butyl alcohol at room temperature for 1 h. Tert-butyl alcohol was replaced with fresh tert-butyl alcohol every 15 min. The gelatin microfibers were allowed to stand at 4 °C for 12 h to solidify tert-butyl alcohol (freezing point of approximately 26 °C). The gelatin microfibers were vacuum-dried to obtain solid microfibers (Figure 1b). Gelatin microfibers encapsulating fluorescent particles were fabricated with an aqueous gelatin solution suspending fluorescent microspheres (diameter: 1 μm) (Fluoresbrite^®^ YG carboxylate microspheres, Polysciences, Warrington, PA, USA) using the same procedure described above. Treatment with citrate and freeze-drying of fibers were conducted in more than ten independent experimental batches.

### 2.3. Fabrication of Capillary-Sized Microchannel Networks in Collagen Gels

An acrylic box (internal dimensions: 30 × 25 × 8 mm) equipped with four short injection needles (inner diameter: 840 μm) was designed in our laboratory (Figure 1c). The needles were cut at 90° to their axial direction. Two long stainless-steel needles (outer diameter: 700 μm) were passed through the short needles fixed to the box. The gelatin microfibers were placed in the center of the acrylic box in contact with the two long needles penetrating the box. The gelatin microfibers were wetted with ethanol by filling the acrylic box with 99% (*v*/*v*) ethanol (4 °C). Next, 99% (*v*/*v*) ethanol was replaced with distilled water (4 °C). Subsequently, distilled water was replaced with a type I collagen solution (2.4 mg/mL, 4 °C) (Figure 1c). The collagen solution (2.4 mg/mL) was prepared by mixing 8 volumes of a stock solution (3.0 mg/mL porcine type I collagen) (Cellmatrix Type I-A, Nitta Gelatin, Osaka, Japan) with 1 volume of an aqueous solution (0.05 N NaOH, 200 mM HEPES, and 22 mg/mL NaHCO_3_) and 1 volume of 10 × minimum essential medium. The box was placed in an incubator (20 °C) for 20 min to allow gelation of the collagen solution and then placed in the other incubator (37 °C) for 20 min to dissolve the gelatin microfibers (Figure 1d). Subsequently, the two long needles were withdrawn from the box. After capping one of the four short needles fixed to the acrylic box, heparinized rat blood was manually injected at 0.3 mL/min (Figure 1e).

After the collagen gel was incubated at 37 °C for 20 min, the region of the gel where the gelatin microfibers had been positioned was embedded in paraffin. Paraffin embedding was performed in accordance with standard histopathological procedures for biological tissues. The paraffin-embedded collagen gel was sectioned into slices with a thickness of 15 μm and observed using scanning electron microscopy (SEM) (S-3000N, Hitachi, Tokyo, Japan). All procedures, including microfiber embedding in collagen gels, heparinized rat blood perfusion, and SEM observation of paraffin-embedded gel slices, were conducted in three independent experimental batches.

## 3. Results and Discussion

### 3.1. Fabrication of Gelatin Microfibers

In this study, we aimed to fabricate capillary-sized microchannels within a collagen gel by dissolving gelatin microfibers with a diameter of approximately 10 μm—identical to that of native capillaries—through an increase in the temperature to 37 °C (Figure 1c,d). To achieve temperature-induced dissolution of the gelatin microfibers within the collagen gel, the microfibers must be stabilized via reversible physical crosslinking rather than chemical crosslinking involving covalent bond formation. Physical crosslinking of gelatin molecules is typically achieved through either the (i) formation of an α-helix structure upon cooling of aqueous gelatin or (ii) evaporation of water from the gelatin solution. Using the electrospinning technique, fine gelatin fibers can be fabricated by evaporating water from an elongated jet of aqueous gelatin [31]. However, while this method is highly suitable for fabricating nano-sized fibers, it is extremely difficult to fabricate 3D fibers with a diameter of approximately 10 μm. In contrast, wet spinning has the potential to fabricate these microfibers. Therefore, we initially attempted to extrude aqueous gelatin (80 °C) from a single cylinder into an immiscible liquid paraffin bath at 4 °C; however, because thermal gelation of the gelatin solution required several seconds of cooling, we could not obtain continuous fiber-shaped gelatin hydrogels. Hence, we attempted to fabricate gelatin hydrogel microfibers of approximately 10 μm in diameter by employing our unique wet-spinning technique [29]. In this method, the formed Ca-Alg hydrogel shell protects the internal gelatin solution and provides sufficient time for it to undergo thermal gelation via cooling, yielding gelatin hydrogel fibers within the Ca-Alg hydrogel fibers. When the fibers were fabricated using aqueous gelatin suspending fluorescent particles, no flow of the fluorescent particles was observed within the gelatin solution in the gelatin/Ca-Alg fibers, indicating successful gelation of the gelatin solution inside the Ca-Alg hydrogel fibers.

The gelatin solution was prepared and extruded at 80 °C to prevent premature thermal gelation inside the syringe barrel, as the physical gelation temperature of 10% (*w*/*v*) gelatin is around 40 °C. Although thermal exposure at 80 °C could potentially induce partial hydrolysis or a reduction in the molecular weight of gelatin chains, even with the 2.5 h exposure duration set for fiber preparation, physical gelation was successfully achieved upon cooling in the 4 °C bath without compromising structural fiber formation.

**Figure 2:**
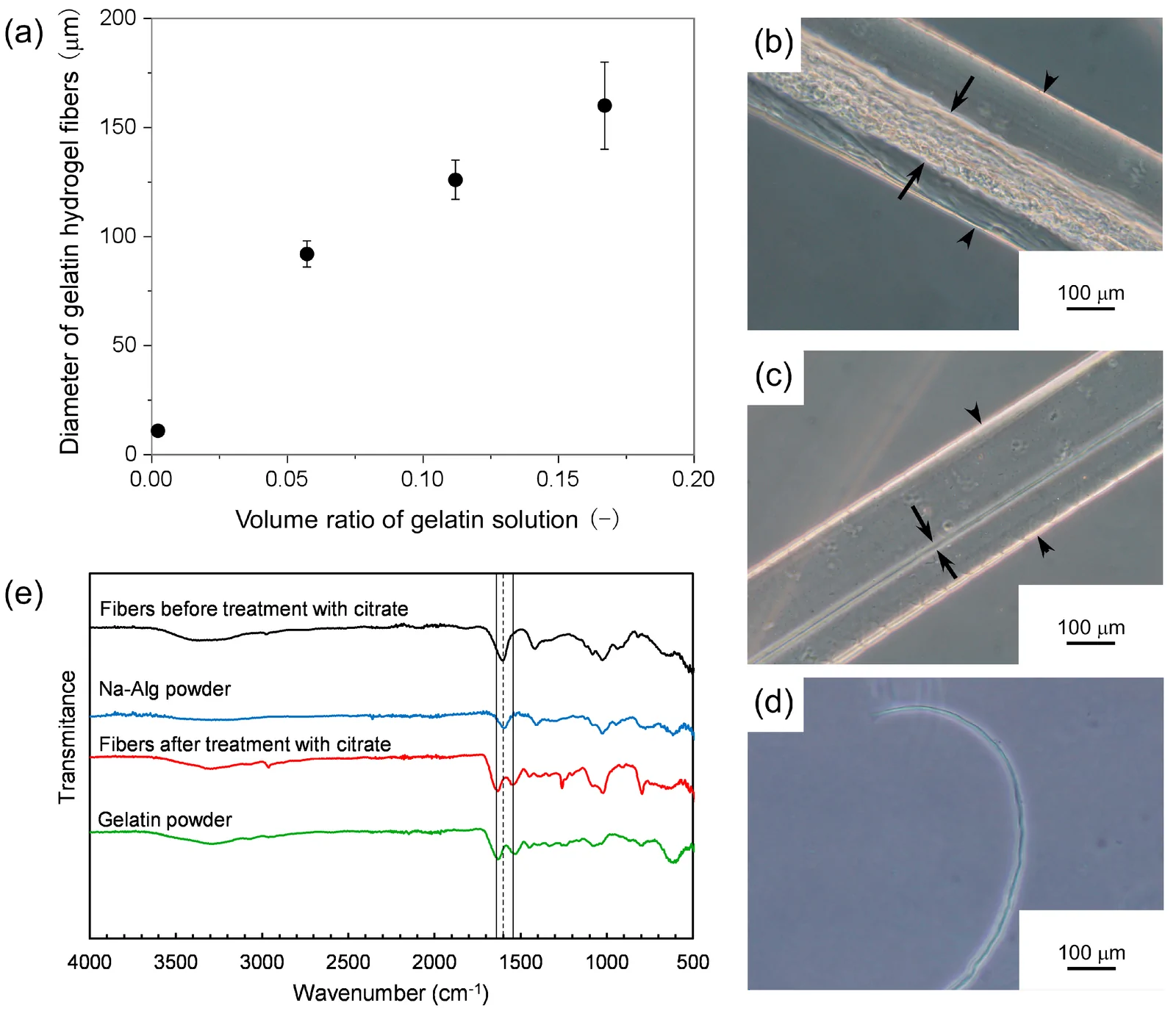
(**a**) Relationship between the volume ratio of gelatin solution and diameter of gelatin hydrogel fibers formed inside Ca-Alg hydrogel fibers (*n* = 50). Data are presented as mean ± standard deviation. (**b**,**c**) The gelatin/Ca-Alg hydrogel fibers prepared at the volume ratios of 0.11200 (**b**) and 0.00225 (**c**). Arrows and arrowheads show gelatin and Ca-Alg hydrogel fibers, respectively. (**d**) Fibers after treatment with citrate. (**e**) FT-IR spectra of fibers before and after treatment with citrate.

**Figure 3:**
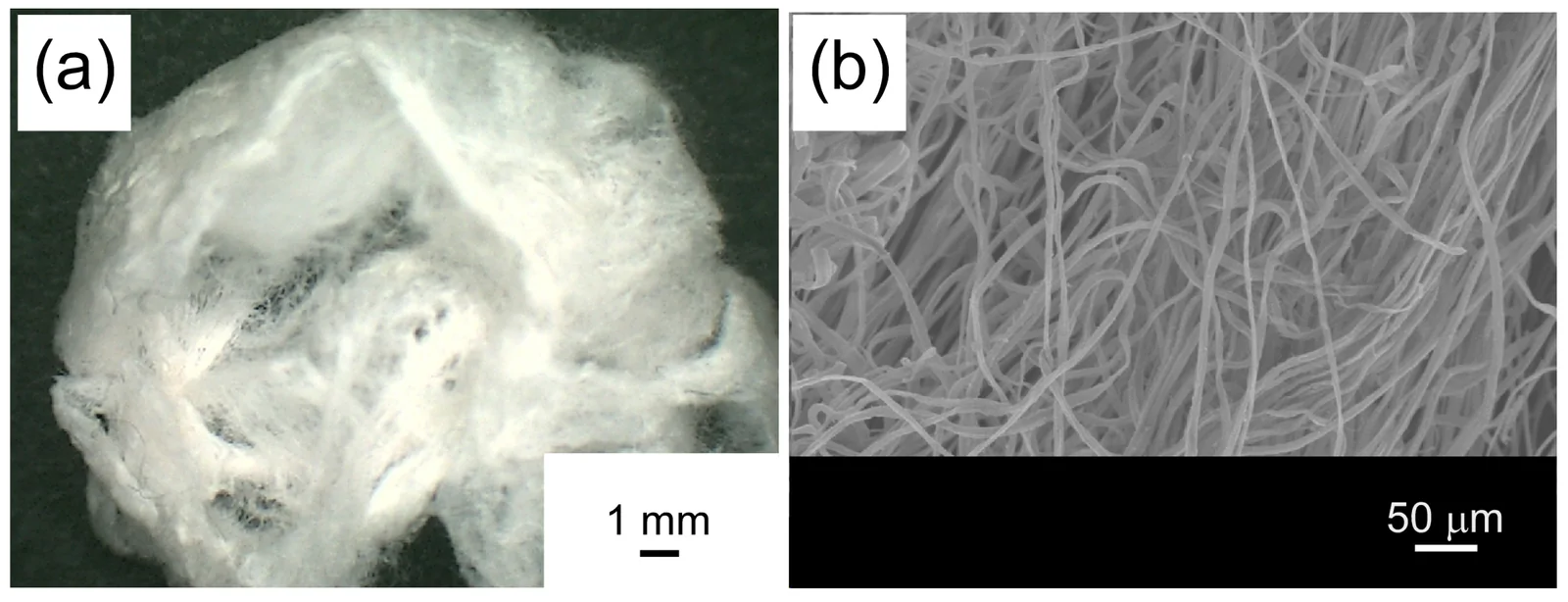
(**a**,**b**) Appearance of gelatin microfibers (**a**) and SEM image of the microfibers (**b**).

Next, to obtain gelatin microfibers with a diameter of approximately 10 μm, the relationship between the volume ratio of the gelatin solution and the diameter of the gelatin fibers was investigated (Figure 2a–c). The diameter of the gelatin hydrogel fibers decreased as the volume ratio of the gelatin solution decreased, yielding gelatin hydrogel fibers with a diameter of 11 ± 2 μm at the volume ratio of 0.00225. After treatment with a trisodium citrate aqueous solution, we visually confirmed that Ca-Alg hydrogel was removed from the gelatin/Ca-Alg hydrogel fibers and that there was no difference in the diameter of the gelatin microfibers before and after the treatment (11 ± 1 μm) (Figure 2d). FT-IR analysis shows that a peak attributable to the asymmetric stretching vibration of COO^−^ groups in alginate was observed at 1600 cm^−1^ in the gelatin/Ca-Alg fibers (Figure 2e). After the treatment with trisodium citrate, the peak was not observed. Alternatively, peaks attributable to the C=O stretching vibration of amide I at 1632 cm^−1^ and the N–H bending and C–N stretching vibrations of amide II at 1547 cm^−1^ were observed in the samples. These results show that Ca-Alg hydrogel was successfully removed from gelatin/Ca-Alg hydrogel fibers. Figure 3 shows 3D solid gelatin microfibers after Ca-Alg hydrogel removal and subsequent freeze-drying, confirming that these two processes did not affect the diameter of the gelatin microfibers (diameter after freeze-drying: 10 ± 2 μm).

### 3.2. Microchannel Formation via Dissolution of Gelatin Microfibers in Collagen Gels

To visually evaluate whether open microchannels could be fabricated in a collagen gel using gelatin microfibers via the procedure illustrated in Figure 1, we first attempted microchannel formation using a single gelatin microfiber. The single gelatin microfiber was first incubated within the collagen solution at 20 °C for 20 min (gelation of the solution) and then incubated within the gel at 37 °C for 20 min (dissolution of the microfiber). Observation of the microfiber region confirmed that the encapsulated fluorescent particles actively flowed (Video S1). This demonstrates that a microchannel can be successfully fabricated within a collagen gel using the procedure shown in Figure 1. We confirmed that the microchannel (12 ± 1 μm) had a similar diameter to the gelatin microfiber.

**Figure 4:**
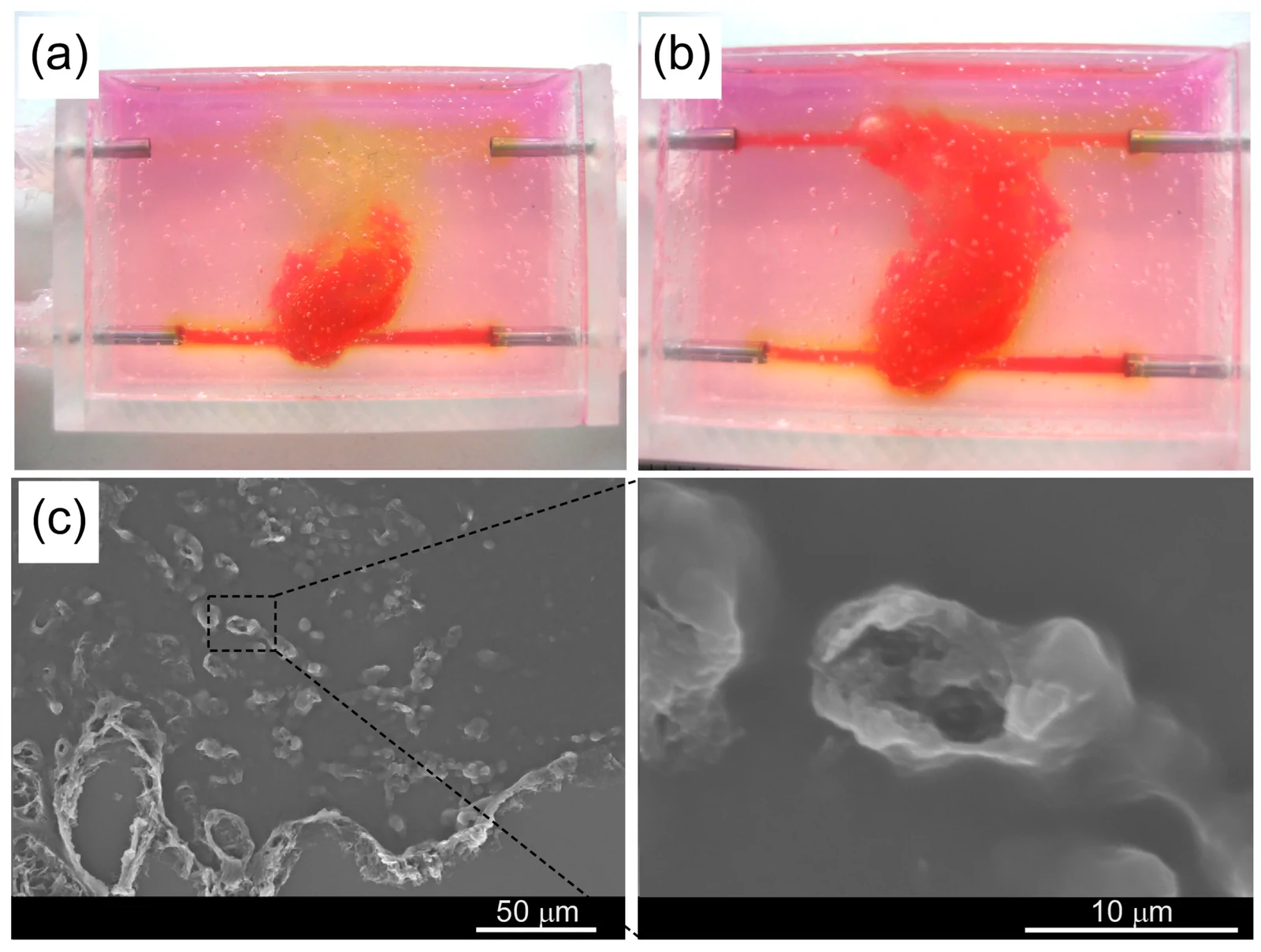
(**a**,**b**) Progressive infusion of heparinized rat blood into channels formed by sacrificial gelatin microfibers. (**c**) A cross-section of a thin slice of the collagen gel at the site where the gelatin microfibers had been embedded.

Next, we investigated whether 3D microchannels could be fabricated using the gelatin microfibers. When heparinized rat blood was slowly infused into the inlet channel generated by withdrawing the long stainless-steel needle from the collagen gel, the injected blood ascended against gravity through the region where the gelatin microfibers had been embedded, successfully reaching the outlet channel formed by the withdrawal of the second long stainless-steel needle (Figure 4a,b). Furthermore, cross-sectional observation of a thin slice of the collagen gel at the site where the gelatin microfibers had been embedded revealed clear cross-sections of the microchannels (approximately 10 μm in diameter), although larger channels resulting from microfiber aggregation were also observed (Figure 4c). These results indicate that capillary-sized microchannels can be fabricated inside a collagen gel matrix, which is challenging to achieve through conventional sacrificial LCST microfiber templating due to thermodynamic mismatch during cold collagen preparation.

Comparing the dimensions achieved in this work (10–11 µm fiber diameter and 10–12 µm channel diameter) with existing literature shows the capability of our method. Most conventional top-down strategies, including 3D bioprinting, sacrificial carbohydrate glass printing/templates, and molded Pluronic templates, typically yield microchannels with diameters ranging from 100 to 500 µm, which correspond to arterioles or venules rather than native capillaries [4,5,6,7,17,18,19,20]. While electrospun LCST microfibers (e.g., PNIPAM) can achieve smaller channels (~10–50 µm), they remain thermodynamically incompatible with type I collagen hydrogels [14,15]. Although hydrophobic polymer (such as poly(methyl methacrylate)) microfibers can produce 10–20 µm channels in synthetic or non-collagen matrices [16], template removal requires cytotoxic organic solvents. Therefore, our UCST gelatin microfiber approach enables the rapid fabrication of capillary-sized microchannels (10–12 µm) directly within natural collagen hydrogels under completely aqueous, cell-friendly conditions.

As described above, not only capillary-sized microchannels but also larger channels resulting from microfiber aggregation were observed (Figure 4c). Such aggregation represents a current limitation of this manual embedding process, as it reduces network homogeneity and structural reproducibility. In future studies, the fiber aggregation could be minimized by surface modification (such as plasma treatment to increase the wettability of fiber surfaces) or by controlled alignment during hydrogel casting [14]. Furthermore, explicit quantitative characterizations of the network architecture—such as channel density, length distribution, 3D connectivity, and pressure drop during perfusion—were not fully evaluated in this preliminary Communication and remain important limitations to be addressed in future work.

Additionally, it should also be emphasized that while blood perfusion visually confirms physical channel patency and fluidic continuity, it should not be interpreted as evidence of biological functionality, endothelialization, or true physiological vascularization. The absence of in vitro cell culture and biological validation represents another key limitation of this preliminary Communication. Future studies will incorporate endothelial cell lining, parenchymal cell co-culture, and long-term functional evaluation to establish biological vascularization.

## 4. Conclusions

As preliminary proof-of-concept, we demonstrated the technical feasibility of rapidly fabricating capillary-sized microchannels within a collagen hydrogel using physically crosslinked gelatin microfibers as a thermal sacrificial microfiber template. By coordinating the thermal gelation of collagen (20 °C) and the thermal dissolution of gelatin microfibers (37 °C), we established open microchannels with capillary-sized dimensions (approximately 10 μm in diameter) within 1 h. The present work provides a promising technical foundation for biofabrication in tissue engineering, although current limitations—including microfiber aggregation leading to larger channel dimensions and the lack of comprehensive 3D quantitative characterization and biological evaluations—should be addressed in future studies.

## 5. Patents

The core fabrication strategy for the microchannel structures described in this manuscript is based on the technology disclosed in the following registered patent: “Method for Producing a Microchannel Structure Having a Capillary-like Microchannel Network” (Japanese Patent No. 6782002, Registered on 21 October 2020; Inventors: Takayuki Takei, etc.).

## Data Availability

The original contributions presented in this study are included in the article. Further inquiries can be directed to the corresponding author.

## Supplementary Materials

The following supporting information can be downloaded at https://www.mdpi.com/article/10.3390/ma19173630/s1, Video S1: Dissolution of a single gelatin microfiber in a collagen gel demonstrated by active flow of fluorescence microparticles that had been encapsulated in the microfiber.

## References

[1] Shi J., Votruba A.R., Farokhzad O.C., Langer R. (2010). Nanotechnology in drug delivery and tissue engineering: From discovery to applications. Nano Lett..

[2] Ko I.K., Iwata H. (2001). An approach to constructing three-dimensional tissue. Ann. N. Y. Acad. Sci..

[3] Ochoa E.R., Vacanti J.P. (2002). An overview of the pathology and approaches to tissue engineering. Ann. N. Y. Acad. Sci..

[4] Takei T., Sakai S., Yokonuma T., Ijima H., Kawakami K. (2007). Fabrication of artificial endothelialized tubes with predetermined three-dimensional configuration from flexible cell-enclosing alginate fibers. Biotechnol. Prog..

[5] Barrs R.W., Jia J., Silver S.E., Yost M., Mei Y. (2020). Biomaterials for bioprinting microvasculature. Chem. Rev..

[6] Takei T., Sakai S., Yoshida M. (2016). In vitro formation of vascular-like networks using hydrogels. J. Biosci. Bioeng..

[7] Neumann T., Nicholson B.S., Sanders J.E. (2003). Tissue engineering of perfused microvessels. Microvasc. Res..

[8] Bettinger C.J., Cyr K.M., Matsumoto A., Kaplan D.L., Borenstein J.T., Langer R., Weinberg E.J. (2007). Silk fibroin microfluidic devices. Adv. Mater..

[9] Choi N.W., Cabodi M., Held B., Gleghorn J.P., Stroock A.D. (2007). Microfluidic scaffolds for tissue engineering. Nat. Mater..

[10] Bettinger C.J. (2009). Synthesis and microfabrication of biomaterials for soft-tissue engineering. Pure Appl. Chem..

[11] Cabodi M., Choi N.W., Gleghorn J.P., Lee C.S.D., Bonassar L.J., Stroock A.D. (2005). A microfluidic biomaterial. J. Am. Chem. Soc..

[12] Bettinger C.J., Weinberg E.J., Kulig K.M., Vacanti J.P., Wang Y., Borenstein J.T., Langer R. (2006). Three-dimensional microfluidic tissue-engineering scaffolds using a flexible biodegradable polymer. Adv. Mater..

[13] Huang J.H., Kim J., Agrawal N., Sudarson A.P., Maxim J.E., Jayaraman A., Ugaz V.M. (2009). Rapid fabrication of bio-inspired 3D microfluidic vascular networks. Adv. Mater..

[14] Rector J.A., McBride L., Weber C.M., Grossman K., Sorets A., Ventura-Antunes L., Holtz I., Young K., Schrag M., Lippmann E.S. (2025). Fabrication of endothelialized capillary-like microchannel networks using sacrificial thermoresponsive microfibers. Biofabrication.

[15] Lee J.B., Wang X., Faley S., Baer B., Balikov D.A., Sung H.-J., Bellan L.M. (2016). Development of 3D microvascular networks within gelatin hydrogels using thermoresponsive sacrificial microfibers. Adv. Healthc. Mater..

[16] Takei T., Kishihara N., Ijima H., Kawakami K. (2012). Fabrication of capillary-like network in a matrix of water-soluble polymer using poly(methyl methacrylate) microfibers. Artif. Cells Blood Substit. Biotechnol..

[17] Kinstlinger I.S., Saxton S.H., Calderon G.A., Ruiz K.V., Yalacki D.R., Deme P.R., Rosenkrantz J.E., Louis-Rosenberg J.D., Johansson F., Janson K.D. (2021). Generation of model tissues with dendritic vascular networks via sacrificial laser-sintered carbohydrate templates. Nat. Biomed. Eng..

[18] Shimizu A., Goh W.H., Itai S., Hashimoto M., Miura S., Onoe H. (2020). ECM-based microchannel for culturing in vitro vascular tissues with simultaneous perfusion and stretch. Lab Chip.

[19] Zhou T., NajafiKhoshnoo S., Esfandyarpour R., Kulinsky L. (2023). Dissolvable calcium alginate microfibers produced via immersed microfluidic spinning. Micromachines.

[20] Zhao N., Kulkarni S., Zhang S., Linville R.M., Chung T.D., Guo Z., Jamieson J.J., Norman D., Liang L., Pessell A.F. (2023). Modeling angiogenesis in the human brain in a tissue-engineered post-capillary venule. Angiogenesis.

[21] Zheng F., Derby B., Wong J. (2021). Fabrication of microvascular constructs using high resolution electrohydrodynamic inkjet printing. Biofabrication.

[22] Han J.H., Ko U.H., Kim H.J., Kim S., Jeon J.S., Shin J.H. (2021). Electrospun microvasculature for rapid vascular network restoration. J. Tissue Eng. Regen. Med..

[23] Qassab M.A., Merheb M., Sayadi S., Salloum P., Dabbousi Z., Bayeh A., Harb F., Azar S., Ghadieh H.E. (2025). Organ-specific strategies in bioprinting: Addressing translational challenges in the heart, liver, kidney, and pancreas. J. Funct. Biomater..

[24] Folkman J., Haudenschild C. (1980). Angiogenesis in vitro. Nature.

[25] Black A.F., Berthod F., L’Heureux N., Germain L., Auger F.A. (1998). In vitro reconstruction of a human capillary-like network in a tissue-engineered skin equivalent. FASEB J..

[26] Montesano R., Orci L., Vassalli P. (1983). In vitro rapid organization of endothelial cells into capillary-like networks is promoted by collagen matrices. J. Cell Biol..

[27] Vernon R.B., Sage E.H. (1999). A novel, quantitative model for study of endothelial cell migration and sprout formation within three-dimensional collagen matrices. Microvasc. Res..

[28] Supp D.M., Wilson-Landy K., Boyce S.T. (2002). Human dermal microvascular endothelial cells form vascular analogs in cultured skin substitutes after grafting to athymic mice. FASEB J..

[29] Takei T., Kishihara N., Sakai S., Kawakami K. (2010). Novel technique to control inner and outer diameter of calcium-alginate hydrogel hollow microfibers, and immobilization of mammalian cells. Biochem. Eng. J..

[30] Sakai S., Ono T., Ijima H., Kawakami K. (2002). In vitro and in vivo evaluation of alginate/sol–gel synthesized aminopropyl-silicate/alginate membrane for bioartificial pancreas. Biomaterials.

[31] Khan R., Haider S., Wahit M.U., Haider A., Aqif M., Khan S.U., Yadav R., Razak S.I.A. (2026). Advanced electrospinning techniques for the fabrication of multi-layered 3D nanofiber scaffolds for tissue engineering. Int. J. Biol. Macromol..

